# Potentially Inappropriate Medication among Older Patients Who Are Frequent Users of Outpatient Services

**DOI:** 10.3390/ijerph18030985

**Published:** 2021-01-22

**Authors:** Kung-Chuan Hsu, Hai-Lin Lu, Chi-Ming Kuan, Jin-Song Wu, Chyn-Liang Huang, Pu-Hua Lin, Damien Trezise, Tzu-Chueh Wang

**Affiliations:** 1 Giraffe Pharmacy, Tainan City 710, Taiwan; gighv206@gmail.com; 2 Department of Information Management, Chia-Nan University of Pharmacy and Science, Tainan City 717, Taiwan; hllu2719@gmail.com (H.-L.L.); joyce@mail.cnu.edu.tw (C.-M.K.); 3 National Health Insurance Administration-Kaoping Division, Ministry of Health And Welfare, Kaohsiung City 801, Taiwan; song@nhi.gov.tw; 4 Division of Medicinal Products, Taiwan Food and Drug Administration, Ministry of Health and Welfare, Taipei City 115, Taiwan; clhuang@fda.gov.tw (C.-L.H.); terlanks@fda.gov.tw (P.-H.L.); 5 Department of Applied Foreign Languages, Chia-Nan University of Pharmacy and Science, Tainan City 717, Taiwan; 6 Department of Pharmacy, Chia-Nan University of Pharmacy and Science, Tainan City 717, Taiwan

**Keywords:** older adults care, polypharmacy, potentially inappropriate medication

## Abstract

Aging is accompanied by changes in organ degeneration, and susceptibility to multiple diseases, leading to the frequent occurrence of adverse drug reactions resulting from polypharmacy (PP) and potentially inappropriate medications (PIM) in older patients. This study employs a retrospective cohort design and investigates the association of PP with PIM among older patients with high rates of medical utilization. Using records from a national pharmaceutical care database, an experimental group is formed from patients meeting these criteria, who are then offered home pharmaceutical care. Correspondingly, a control group is formed by identifying older patients with regular levels of use of medical services who had been dispensed medications at community pharmacies. Multivariate logistic regression is performed to assess the association between the rate of PIM and variables, including age, gender, and PP. The study finds that experimental PP participants had a higher rate of PIM prescription (odds ratio (OR) = 5.4) than non-PP control participants (all *p* < 0.001). In clinical practice, additional caution is required to avoid PIMs. Patients engaged in continuously using long-term medication should take precautions in daily life to alleviate related discomforts. Pharmacists should serve as a bridge between patients and physicians to enhance their health and improve their quality of life.

## 1. Introduction

Polypharmacy (PP) is defined as the co-prescribing of multiple medications or concurrent use of more than five drugs [1,2,3,4,5]. Studies have found a positive correlation between patients experiencing PP and higher age, lower levels of education, and female gender [1]. PP can increase the risk of falls in patients, adverse drug reactions (ADR) caused by drug interaction, medication non-compliance, hospitalization, and damage to physiological capacity [1,6,7,8,9,10]. Thus, ADRs may be caused by additional drugs to treat adverse effects from another medication, or lack of strong evidence to discontinue certain drugs [1].

In March 2018, the proportion of the population aged over 65 in Taiwan reached 14%, officially making it an aged society, and it is expected that by 2026 the proportion of older adults will exceed 20%, making Taiwan a super-aged society [11]. Aging leads to changes in the biochemical composition of tissues and organ degeneration, increased susceptibility to multiple diseases, and increased risk of PP and associated ADRs, all of which will affect the care and/or the quality of life of older adults [12,13,14]. Thus, appropriate tools to assess potentially inappropriate medication (PIM) in older adults are required. The use of PIM can lead to predictable ADRs among older people, the criteria for which can be classified into two categories, according to the method of evaluation: Implicit criteria, which are judgment-based quality indicators that focus primarily on the patient rather than drugs or diseases, such as the Medication Appropriateness Index [15,16]; and explicit criteria, focusing on specific medication/medication class [17]. Commonly applied explicit criteria include screening tool of older people’s prescriptions and screening tool to alert to right treatment criteria (STOPP/START criteria) and Beers Criteria [17]. A local scale known as PIM Taiwan has also been developed in Taiwan [18]. Research in the field has found that factors, including female gender, age group under 85, occurrence of PP, and history of myocardial infarction or heart failure are most often associated with PIM [14,19,20,21].

Studies in the United States indicate that PP accounts for about 40% of drug-related problems (DRPs) [22] and is the second most likely cause of therapeutic risk to patients [1]. Studies in Taiwan have reported that 81% of older citizens are prescribed more than five drugs for long-term use, and 38% have engaged in long-term use of more than ten drugs [23]. Due to the high incidence of PIM in older people, DRPs, such as adverse reactions or physical discomfort, may occur [24]. Pharmacists can find various DRPs in patients and improve patient medication adherence and knowledge, particularly when an intervention goes beyond the dispensing of medications in community pharmacies to include home pharmaceutical care [25].

In 2010, the Taiwan Pharmacist Association launched a pharmaceutical home care service. Pharmacists were asked to provide pharmaceutical home care for specific groups to provide early detection of apparent or potential DRPs in patients; communicate and coordinate with physicians and families to facilitate drug education; alleviate the discomfort caused by ADRs through medication reconciliation; and create an environment of trust around the use of medications in patients. If a case met the criteria for the high rate of utilization of medication, the patient would be eligible to receive home healthcare assistance from a pharmacist.

This study aimed to investigate the effects of such home pharmaceutical care on the incidence of PIM among older patients with high rates of medical service utilization, as well as possible associations between the presence or absence of PP among these patients and PIM. It was predicted that patients with high medical utilization would have more PIM drug prescriptions and PP prescriptions (more than five medications) than those with regular utilization. It was further hypothesized that when the population with a high drug utilization rate received home medication care, the incidence of PIM could be reduced.

## 2. Materials and Methods

### 2.1. Study Design

This study employed a retrospective cohort design to investigate differences in the incidence of PIM and polypharmacy between an experimental and control group of older patients using drugs to treat chronic diseases. The experimental group had high rates of utilization of medication and were frequent users of outpatient services, while the control group were not. The experimental group consisted of patients who had attended more than 90 consultations in hospital outpatient services or general practitioner clinics in the previous year, and were either being treated for more than two types of chronic diseases or had been prescribed five or more drugs in more than half of their prescriptions in the previous year [26].

In the study, cases were selected from the database of the Taiwan Pharmacist Association. This database contains two sections: Records of home pharmaceutical care, and records of community pharmaceutical care. The records of pharmaceutical care in community pharmacies were used to identify the control group. Traditionally, after seeing a doctor, patients in Taiwan can go to a local community pharmacy to have their prescriptions filled. Here the pharmacist dispenses the medications and provides pharmaceutical care. Pharmacists can use the patient’s National Health Insurance (NHI) IC Card (smart card) to log in to the NHI-PharmaCloud database. This enables them to view medications the patient has used in the last three months, and ascertain whether there are any potential interactions or compatibility contraindications between new medications and their latest prescriptions [27]. For the purposes of this study, only records relating to patients over 65 years of age who had taken courses of medicine for 28 days or more in the past year and were not frequent users of outpatient services were used to form the control group. The data for this group were obtained from the pharmacy dispensing records in the database, which included age, gender, and medication records of people receiving pharmaceutical care in community pharmacies from 2013 to 2015 [28]. The cases selected had received pharmaceutical care only from community pharmacies, including the judgment of the suitability of medications, interaction effects, and superfluous prescription, and had not received any additional pharmaceutical care. The control group consisted of ‘regular’ older members of the community, and were chosen to provide a basis for comparison with the experimental group.

Patients selected for the experimental group were sampled from frequent users of outpatient services. It was possible that they had received medical care from multiple institutions, and were selected from all over Taiwan based on the above criteria. They were matched with community pharmacies close to their homes, and with their consent, the pharmacists undertook home pharmaceutical healthcare. During the year of the study, a single pharmacist visited the case at home once a month, for a maximum of eight visits. No fees were charged for this service. During each visit, the pharmacist inspected the patient’s medication adherence, their home environment, the medications prescribed by different medical institutions, and whether there were cases of potentially inappropriate medication. Pharmacists were trained in pharmaceutical home care service by the Taiwan Pharmacist Association before the start of this study. After the training, they were considered to have the professional skills required to provide individualized pharmaceutical home care. After conducting a home visit, pharmacists were required to assess and record information about the lifestyle of the participant, along with their informed and actual medications, and provide a systematic assessment of the patient according to the visit record form designed by the Taiwan Pharmacist Association. The pharmacist then filed the record form. Through visits to patients’ homes, pharmacists were able to understand the actual situation of their medication use, discover related problems and implement measures for resolving them.

After obtaining data from the two groups, this study set out to analyze the incidence of PIM in older patients (aged 65 and above) taking medication for chronic diseases. To prevent errors caused by differences in the two samples, the control group was resampled, so that age group and gender proportions within the two samples were equivalent. In addition, cases whose records were incomplete were excluded from the experimental group. The data analysis of this study was approved by the Institutional Review Board of National Cheng Kung University Hospital (B-ER-107-142).

### 2.2. Data Analysis

Baseline characteristics of age and gender were obtained in both experimental and control groups. Age was classified into three categories: 65–74, 75–84, and 85 years and above. The number of drugs prescribed was used to derive categories of PP (taking five or more drugs) and non-PP (taking fewer than five drugs). PIMs were assessed using the Beers Criteria 2015 edition [29]. In the data for the experimental group, the medications recorded on the first visit were defined as medication status before the intervention. The medications being used at the time of the last visit were the final result of multiple episodes of home counseling, and were thus defined as medication status after the intervention.

Related studies have found that myocardial infarction or heart failure were the health conditions most commonly associated with PIM [20]. However, because disease diagnoses are not recorded in the pharmaceutical database, medications prescribed by hospital cardiology departments were chosen to be analyzed for the incidence of PIM, and evaluation of possible related dangers.

Statistical analyses were performed using STATA 16.1 (Stata Corp LLC, College Station, TX, USA). Pearson’s chi-squared test was performed to analyze the difference of PIM and PP between the two groups, with statistically significant differences indicated by *p* < 0.05. Multivariate logistic regression was performed to assess whether PIM had been prescribed more to patients in the experimental group than to those in the control group, we test the interaction between PP and the group. The dependent variable was presence or absence of PIM, and the independent variables were age group, gender, and the interaction between presence or absence of PP and the groups. The time windows in which PIM is measured from all records in the database. To detect the effectiveness of pharmaceutical home care, multivariate logistic regression was performed to assess the prescription rate of PIM in patients in the experimental group. The dependent variable was the presence of PIM prescriptions, and the independent variables were the time of prescription—before the intervention (at the time of the first visit) and after the intervention (at the time of the last visit), age group, and gender. The prevalence of PP was defined as the proportion of the population with PP presence. The prevalence of PP/PIM was defined as the proportion of the population that had at any time in the sample period concurrently been issued PP/PIM prescriptions.

## 3. Results

### 3.1. Baseline Characteristics of Study Participants

In this study, 20,700 patients made up the control group, and 2035 individuals were included in the experimental group. There were 10,250 females in the control group (49.5%) and 1006 females in the experimental group (49.4%). Patients 85 years of age or above formed the smallest age group, and there were no significant statistical differences between the two groups in terms of age or gender distribution (*p* > 0.05). The prevalence of PP was significantly higher in the control group at 62.4% compared with 43.2% in the experimental group (*p* < 0.001). Only 48.8% of patients in the control group had received at least one PIM prescription, compared with about 80% in the experimental group (*p* < 0.001) (Table 1). Overall, there was a lower presence of PP among cases in the experimental group than in the control group, but a higher proportion of this group had been prescribed PIMs.

**Figure 1:**
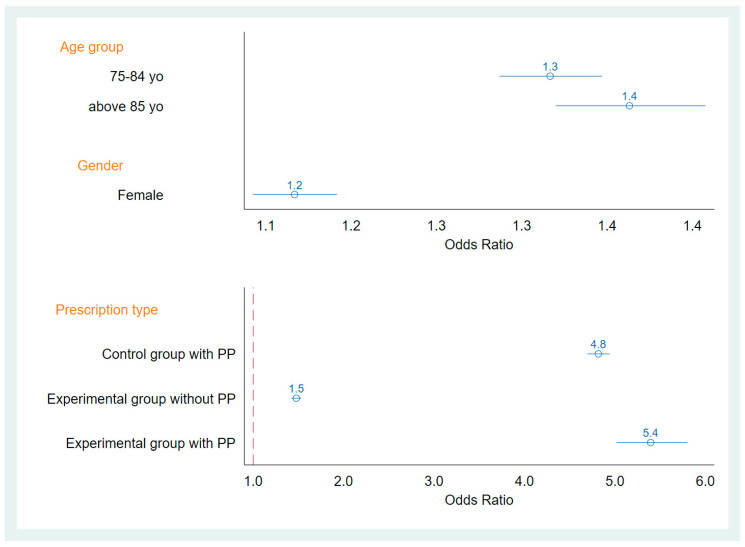
The odds ratio (OR) of PIM prescription rates in PP. yo = years old.

Multivariate logistic regression analysis of PIM prescription among the interaction between patients in the experimental group compared to those in the control group and the presence or absence of PP. The odds ratio (OR) of PP participants in the experimental group receiving a PIM prescription was 5.4 (95% confidence interval: 5.02–5.80) compared to the non-PP participants in the control group. By comparison, among the PP patients in both groups, the experimental group had a 1.125-fold higher prescription rate of PIM than those in the control group (Figure 1). After pharmacist interventions, the PIM prescription OR was 0.8 (95% confidence interval: 0.68–0.98) compared with before pharmacist intervention (Table 2).

There is more PIM prescription in female, aged. The OR of PP participants in the experimental group receiving a PIM prescription was 5.4 compared to the non-PP participants in the control group

### 3.2. Prescription Medication Analysis

Medication analysis showed that PIMs accounted for 20.71% of the overall prescriptions in the experimental group; these included alprazolam, the most frequently prescribed PIM, followed by zolpidem, and diclofenac (Table 3). In the control group, the most frequently prescribed PIM was aspirin, followed by alprazolam, mefenamic acid, and zolpidem. In our study, 1136 of the prescriptions in the experimental group were prescribed by the cardiology department, of which 491 were PIM (43.22%); in terms of drug classification, the five most common were alprazolam, dipyridamole, doxazosin, amiodarone, and ticlopidine (Table 4).

## 4. Discussion

In this study, it was found that under similar gender and age distributions, patients who frequently used medical care and those with polypharmacy had higher rates of PIM prescription. In addition, a review of prescriptions containing polypharmacy and PIM found that PP patients in the experimental group had a 1.125-fold (5.4/4.8) higher rate of prescription of PIM than PP patients in the control group. Among the PIMs used, alprazolam was the most frequently prescribed, followed by zolpidem.

The prevalence of PP in this study was 62.4% in the control group, but 43.2% in the experimental group. The prevalence of PP in related studies varies widely, ranging from 46.6% to 93% [8,9,10,21,23,32,33], and the results of our study also reflect this wide range. Ahmed et al. reported that PP was 2.3 times more associated with ADRs [10]. Our study also confirms the results of numerous studies that populations with high utilization of medical care will require more attention regarding the use of medications, because these groups are more likely to be prescribed PIMs [32,34,35,36]. Nightingale et al. also found that patients with PP or multiple diseases were more likely to have PIM prescriptions, due to the poorer physiological state of these patients, which places them at greater risk of being prescribed PIMs [37]. Alhawassi et al. found that women, those with less education, patients with PP, and older people were more likely to encounter ADRs [38]; Hedna et al. revealed that 46.0% of older patients had received at least one PIM prescription [14]. Resink et al. in 2018 found that the risk factors for ADR were PP, age over 85 years, and multiple diseases [1], and the older adult and PP populations were more susceptible to adverse drug reactions; therefore, older people should be more cautious, due to their greater physiological susceptibility [12,13,14]. In this study, nearly 80% of patients in the experimental group had received a PIM prescription, possibly as a result of their higher rates of medical utilization, but more information is needed about the severity of their conditions and the types of medical institutions prescribing the medications for this to be fully understood. Among the cases studied, the experimental group had high rates of medical service utilization, as opposed to the control group, who were regular outpatients. High-frequency patients are usually suffering from more serious illnesses, so it was not unexpected that the experimental group would be more prone to the risk of PIM prescription. For patients with a high frequency of consultations, medical practitioners will generally try to minimize the prescription of multiple medications. It is to be expected that the intervention of a pharmacist will lead to the discovery of PIMs, and the risk of PIM can consequently be reduced. PP participants in this group were also 1.125 times more likely to receive a PIM prescription than the control group. Therefore, compared to the general public, PP participants in the experimental group who had multiple diseases and prescriptions from different medical institutions would have a higher chance of being prescribed PIMs. In the future, when a doctor writes a prescription or a pharmacist dispenses a medication, the patient’s consent will be sought first; then, the National Health Insurance Pharma Cloud System will be consulted for drug interactions, the appropriateness of the current medication for the patient, and the presence of possible risks. Warning signs would include medications, such as: Medications for hypertension, which can lead to orthostatic hypotension and fall-related injuries, and anticholinergics, may cause discomforts, such as dry mouth and constipation [29,30,31].

Related studies have suggested that the drugs that cause ADRs are primarily cardiovascular drugs, followed by central nervous system drugs [38]. In this study, by analyzing the number of drugs prescribed, we found that central nervous system drugs were the most frequently prescribed to participants in the experimental group, followed by cardiovascular drugs. Both types of drugs are prone to cause ADRs, and should be carefully assessed when being dispensed. The most commonly used PIM in the experimental group was alprazolam. Alprazolam is a type of immediate and short-acting benzodiazepine (BZD) with a hypnotic sedative effect. However, aging may enhance the hypnotic effect and increase the risk of cognitive impairment, delirium, falls, fractures, and motor vehicle-related injuries [29]. Clonazepam is a long-acting BZD with highly potent anticholinergic and sedative effects; it can also lead to hypnotic effects. It is not recommended for continuous use for more than four weeks at a time, as this may increase the risk of falls [30], and is contraindicated for older patients who have chronic obstructive pulmonary disease, pneumonia, sleep apnea, urinary incontinence, or who are at risk of falls or fainting [31]. Zolpidem is in the class of non-benzodiazepine (NBZD) hypnotics; it may increase the risk of driving accidents or the risk of falls [30]. Carbinoxamine is a first-generation antihistamine with a high degree of anticholinergic effect with potential side effects, such as lethargy, confusion, dry mouth, and constipation [29]. The relevant criteria recommend switching to a newer generation of antihistamines [30,31]. Diclofenac is a non-steroidal anti-inflammatory drug (NSAID) that may increase the risks of gastrointestinal bleeding or digestive ulcers. While this risk may not be completely avoided, it can be reduced when diclofenac is taken with a proton-pump inhibitor (PPI). The STOPP/START criteria also states that NSAIDs increase the risk of gastric ulcer or gastrointestinal bleeding and recommends that concurrent PPI or H_2_ antagonist be prescribed instead [30].

In addition, the most common medications that cause adverse drug effects are cardiovascular drugs, and patients with cardiovascular diseases, myocardial infarction, or heart failure are most commonly associated with PIM [14,20]. About 40% of the PIM prescriptions found in this study were issued by cardiovascular departments. The most prescribed was alprazolam, followed by dipyridamole, doxazosin, amiodarone, and ticlopidine. Both dipyridamole and ticlopidine are antithrombotic drugs that may cause orthostatic hypotension and have more effective and safer alternatives [31]. It is suggested that they can be replaced with clopidogrel or aspirin, but these drugs should be used with caution in patients who have peptic ulcers and who also require PPIs [30,31]. Doxazosin is a highly selective alpha-1 blocker for peripheral blood vessels and has a higher risk of orthostatic hypotension. Amiodarone is a Class III antiarrhythmic drug, with greater toxicity in those with atrial fibrillation; thus, the effect of the treatment should be considered first [29]. The PIM-Taiwan criteria suggest that doxazosin is not recommended for patients with urinary retention [31], and amiodarone is not recommended as a first-line antiarrhythmic drug [30]. Each drug has its own characteristics for the listing as a PIM. If pharmaceutical care also includes delivery of the recommended medication instructions, the general public will experience less discomfort and be less likely to need emergency medical care. To avoid the discomfort of the PIMs, there should be more in-home pharmaceutical care to detect the ADRs present, and the physician should get more educations in such patients.

Patients in the experimental group had a higher number of PIM prescriptions than those in the control group. This study deliberately selected patients for this group with high medical utilization combined with two types of chronic diseases, those who may have received prescriptions from multiple hospitals, and those with a greater likelihood of PIMs or ADRs. We also analyzed the association between PP and the incidence of PIM and found that, since this population was prone to being prescribed PIMs, which may cause drug-related problems, further study is needed to determine whether home visits by pharmacists can reduce the incidence of PIMs.

### 4.1. Study Limitations

In this study, we analyzed only those patients in the dataset for whom complete information was provided. Some of those who received in-home pharmaceutical care could not be included, since the pharmacist in the community pharmacy failed to include all relevant information in the database entry. For the continuation of this study in the future, the usability of the records may be enhanced by regularly pre-checking the estimated missing relevant information in data after filing by pharmacists and by then automatically sending out notifications when the program detects missing or incorrectly completed fields in the data submitted.

The Beers criteria used to identify PIM in this study were based mainly on clear definitions of medications and associated discomfort. However, additional data, such as creatinine clearance or the PIM prescriptions, were not available for most patients. The data for this study is also missing records of patient illnesses, and data on the values of tests and the types of institutions issuing the prescriptions. Therefore, it is suggested that future studies include relevant biochemical test values or related diagnostics gathered during the home visits of pharmacists, and that the STOPP/START criteria [30] be further used to analyze the differences between criteria. In addition, due to a lack of complete data on the diagnosis of diseases and test results, it was only possible to check the suitability of medications. It was not possible to conduct checks of drug-disease or drug-syndrome interactions in accordance with the Beers Criteria, or to inspect adjustments of medications for renal function. It is also suggested that when conducting pharmaceutical healthcare, it may not always be necessary to regard the guidelines as absolute. Cases need to be assessed according to their individual circumstances, in order to avoid problems caused by overestimations in the standards and ensure the good health of the patient.

It should also be noted that this study adopted the 2015 version of the Beers Criteria, rather than the latest (2019) version [31]. The reason for this was that the new version added glimepiride, methscopolamine, and pyrilamine, but deleted ticlopidine and pentazocine because they are no longer used in the US market [39]. However, in this study, ticlopidine was found to be the fifth most frequently prescribed medication for cardiology patients. Given this relatively high level of usage, the 2015 version of the Beers Criteria was considered to be more suited to the local context.

The PIM-Taiwan guidelines use 13 PIM standards issued in the period from 1991 to 2009 for drug selection, so there is a lack of newer drugs in these guidelines [31]. In 2019, the guidelines were updated in accordance with the eight standards published from 2011 to 2017. The new list includes seven categories with a total of 169 single-component drugs and nine adjuvant drugs [18]. However, as the criteria adopted in our study included nine categories, and the revised PIM-Taiwan guidelines still lack antithrombotics and anti-infective medications, we elected to use the Beers Criteria. In the future, it is suggested that related research compare the prevalence of PIM using both the Beers and Taiwan standards.

### 4.2. Prospects

In the future, we hope to further analyze the DRPs exposed by pharmacists and understand the problems encountered during pharmacist inventions, to provide appropriate suggestions and solutions. We also hope that future research can compare the causes and effects of the incidence of PIM in older patients before and after treatments from different medical departments during pharmacist inventions. Thus, we hope to propose possible solutions according to the risk factors associated with the long-term treatment of patients with PP and PIM. To investigate these problems more rigorously, it is suggested that in future studies, the state of diseases be further examined. When pharmacists record details of pharmaceutical dare, it is recommended they add disease records, such as the ICD code of the doctor’s diagnosis and the medical department responsible for the case.

## 5. Conclusions

To extend the scope of this research, future studies may focus on pharmacists or participants by geographical region or type of urbanization.

This study focused on patients with high rates of medical utilization, as defined by more outpatient visits. Because such patients are at higher risk of being prescribed PIMs, pharmacists should take great care when performing medication reviews in the homes of such patients. PIM cannot always be avoided in older citizens, yet pharmacists should pay attention to the possible side effects of prescriptions in adults with PP and provide special notes during home visits, to reduce discomfort or the need for emergency medical treatment caused by side effects or drug interactions. In addition, pharmacists should work to obtain the trust of patients by providing them with professional and cordial care, and further serve as a bridge between patients and physicians to improve the health, well-being, and quality of life of patients.

## Figures and Tables

**Table 1:** Baseline characteristics of the study participants.

Characteristics	Control Group	Experimental Group	*p*-Value
	*n* = 20,700	*n* = 2035	
Gender			
Male	10,450 (50.5%)	1029 (50.6%)	0.994
Female	10,250 (49.5%)	1006 (49.4%)	
Age group			
65–74 years	8350 (40.3%)	820 (40.3%)	0.999
75–84 years	9000 (43.5%)	885 (43.5%)	
≥85 years	3350 (16.2%)	330 (16.2%)	
Case with PP			
Yes	12,914 (62.4%)	880 (43.2%)	<0.001
No	7786 (36.4%)	1155 (56.8%)	
Case with PIM			
Never prescribed	10,598 (51.2%)	415 (20.4%)	<0.001
Had been prescribed	10,102 (48.8%)	1620 (79.6%)	

PP, polypharmacy; PIM, potentially inappropriate medication.

**Table 2:** The OR of PIM on the experimental group.

Group	Odds Ratio (95% Confidence Interval)
Intervention	
before	Reference group
after	0.8 (0.68–0.98)
Gender	
male	Reference group
female	1.1 (0.91–1.30)
Age group	
65–74	Reference group
75–84	1.0 (0.80–1.18)
above 85	1.0 (0.81–1.35)
Cases with PP	5.8 (5.47–6.11)

**Table 3:** The top 10 most frequently prescribed potentially inappropriate medications and their relation to the relevant criteria.

Composition	Number of Prescriptions	Beers 2015	STOPP/START V2 [30]	PIM-Taiwan [31]
alprazolam	739	V	V	V
zolpidem	647	V	V	V ^+^
diclofenac	618	V	V	V
lorazepam	509	V	V	V
carbinoxamine	483	V	V	V
metoxazone	478	V		
metoclopramide	459	V	V	V ^+^
clonazepam	403	V	V	V
dipyridamole	398	V	V	
scopolamine	360	V	V	V ^+^

V-The criteria indicated the related risks. ^+^ only on 2019 version. STOPP/START: screening tool of older people’s prescriptions and screening tool to alert to right treatment criteria

**Table 4:** The top 10 PIM drugs prescribed by cardiology departments and their relation to the relevant criteria.

Composition	Number of Prescriptions	Beers 2015	STOPP/START V2 [30]	PIM-Taiwan [31]
alprazolam	95	V	V	V
dipyridamole	63	V	V	
doxazosin	56	V	V	V
amiodarone	49	V	V	V
ticlopidine	44	V	V	V ^$^
digoxin	34	V	V	V
zolpidem	28	V	V	V ^+^
lorazepam	26	V	V	V
clonazepam	25	V	V	V
estazolam	21	V	V	V

V-The criteria indicated related risks. ^+^ only in the 2019 version. ^$^ only in the 2012 version.

## Data Availability

Data sharing not applicable.
